# Ventricular septal defect

**DOI:** 10.1186/s13023-014-0144-2

**Published:** 2014-12-19

**Authors:** Diane E Spicer, Hao H Hsu, Jennifer Co-Vu, Robert H Anderson, F Jay Fricker

**Affiliations:** Department of Pediatric Cardiology, University of Florida, Gainesville, Florida USA; Department of Pediatric Cardiology, Children’s Hospital and Medical Center, Omaha, Nebraska USA; Institute of Genetic Medicine, Newcastle University, Newcastle upon Tyne, UK

**Keywords:** Classification, Terminology, Perimembranous, Conoventricular, Conduction tissues

## Abstract

**Background:**

Ventricular septal defects are the commonest congenital cardiac malformations. They can exist in isolation, but are also found as integral components of other cardiac anomalies, such as tetralogy of Fallot, double outlet right ventricle, or common arterial trunk. As yet, there is no agreement on how best to classify such defects, nor even on the curved surface that is taken to represent the defect.

**Methods:**

Based on our previous pathological and clinical experiences, we have reviewed the history of classification of holes between the ventricles. We proposed that the defects are best defined as representing the area of deficient ventricular septation. This then permits the recognition of clinically significant variants according to the anatomic borders, and the way the curved surface representing the area of deficient septation opens into the morphologically right ventricle.

**Results:**

Clinical manifestation depends on the size of the defect, and on the relationship between systemic and pulmonary vascular resistances. Symptoms include failure to thrive, along with the manifestations of the increase in flow of blood to the lungs. Diagnosis can be made by physical examination, but is confirmed by echocardiographic interrogation, which delineates the precise anatomy, and also provides the physiologic information required for optimal clinical decision-making. Cardiac catheterization offers additional information regarding hemodynamics, particularly if there is a concern regarding an increase in pulmonary vascular resistance. Hemodynamic assessment is rarely necessary to make decisions regarding management, although it can be helpful if assessing symptomatic adults with hemodynamically restrictive defects. In infants with defects producing large shunts, surgical closure is now recommended in most instances as soon as symptoms manifest. Only in rare cases is palliative banding of the pulmonary trunk now recommended. Closure with devices inserted on catheters is now the preferred approach for many patients with muscular defects, often using a hybrid procedure. Therapeutic closure should now be anticipated with virtually zero mortality, and with excellent anticipated long-term survival.

**Conclusion:**

Ventricular septal defects are best defined as representing the borders of the area of deficient ventricular septation. An approach on this basis permits recognition of the clinically significant phenotypic variants.

## Introduction

Accurate estimates of the prevalence of holes between the ventricles are difficult to achieve. When patients with bicuspid aortic valves and mitral valvar prolapse are excluded, [1] ventricular septal defects are recognized as being the commonest congenital cardiac malformations [2]. The defects can exist in isolation, can be complicated by additional intracardiac lesions, or can be part of more complex combinations, such as tetralogy of Fallot, double outlet right ventricle, transposition, or functionally univentricular hearts. In this review, we focus on the isolated defect, although the system we describe for classification is valid for all situations in which there is defective ventricular septation [3]. Although it would seem intuitive to define ventricular septal defects as no more than holes within the ventricular septum, the situation is not as clear-cut as might be imagined. Some defects exist in a location where, in the normal heart, there are no ventricular septal structures. Problems also exist in providing a uniform definition for the curved surface taken to represent the defect, which in most instances is non-planar. It is this problem that has underscored some of the differences existing in classification of the phenotypic variants [4]. Other problems in classification reflect the different names given to holes that have the same phenotypic features [5]. We begin our description of our results, therefore, with a discussion of the background underscoring description of the phenotypic variants. Accurate distinction of these variants is essential for correct diagnosis and, when appropriate, therapeutic closure.

## Results

### Definition of the defect

**Figure 1:**
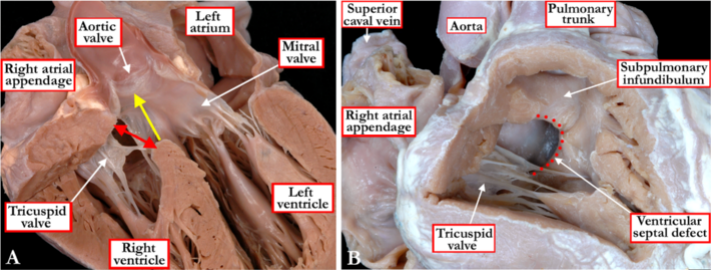
**The images show the problems existing in defining the boundaries of the area of space that represents the “ventricular septal defect”.** Panel **A** shows a simulated five chamber echocardiographic cut in a specimen with overriding of the aortic root relative to the apical muscular septum. The yellow arrow shows the continuation of the long axis of the muscular ventricular septum. This area marks the true geometric interventricular communication, which is almost planar. This virtual plane, however, can never be closed, since its cranial margin is formed by the leaflets of overriding aortic root. The red double-headed arrow shows the margins of the curved surface that would be closed so as to restore septal integrity. Although shown as a planar entity, in reality the surface is markedly curved due to the non-planar configurations of its boundaries. It is shown in planar format for the sake of simplicity. As shown in Panel **B**, it is the margins of this curved surface, outlined by the red dots, that are taken as representing the ventricular septal defect when viewed from the right ventricle. Note that its cranial border is formed by the muscular outlet septum, which is malaligned relative to the apical muscular septum, accentuating the non-planar configuration of its surface.

If defined in simplest of fashions, a ventricular septal defect is no more than a hole between the ventricles. Indeed, in the Romance languages, such as French, Spanish, Portuguese, and Italian, the entity is described as an interventricular communication, rather than a ventricular septal defect [6,7]. In many ways, interventricular communication is a better term, since as already discussed in our introduction, the hole between the ventricles is not always within the confines of the normal ventricular septum. It is difficult, nonetheless, to provide an all-encompassing, and at the same time simple, definition. In part, this is because the boundaries of the hole between the ventricles, and therefore the borders of the curved surface chosen to represent the septal defect, are not always obvious. The easiest of defects to define are those bounded on all margins by the musculature of the ventricular septum. Even in hearts with such muscular defects as viewed from the right ventricle, however, difficulty can arise in determining the plane chosen to represent the defect when there is malalignment between the apical and outlet components of the ventricular septum, and hence some degree of overriding of the leaflets of an arterial valve. Such problems also arise when the defect itself has partly fibrous borders, and the crest of the deficient muscular septum is overridden by the orifice of either an atrioventricular or an arterial valve. In the setting of valvar overriding, therefore, be the overriding valve atrioventricular or arterial, and if we are to provide an all-encompassing definition, a decision must first be made regarding the curved surface which represents the defect. There are two options. The first is to take the basal continuation of the long axis of the ventricular septum as the defect, since this area, also not necessarily planar, is the geometric interventricular communication (Figure 1A) [3]. The alternative is to define the right ventricular boundaries of the entrance to the cone of space subtended between the orifice of the overriding valve and the crest of the muscular ventricular septum (Figure 1B) [8]. It is this right ventricular boundary of the entrance to the subvalvar cone of space that is of most importance clinically. The area is rarely planar, and is best considered as a curved surface. The borders of the curved surface provide the locus around which a surgeon will place a patch so as to tunnel the overriding valvar orifice into the morphologically left ventricle, thus restoring septal integrity in those patients with otherwise concordant atrioventricular and ventriculo-arterial connections. This is also the area across which devices are inserted percutaneously. It is also this curved surface that is the site of final closure of the embryonic interventricular communication [9]. A patch placed along the basal continuation of the long axis of the muscular ventricular septum would transect the leaflets of any overriding valve. This latter locus, therefore, is obviously not the site chosen for therapeutic closure, either surgically or by percutaneous insertion of a device. For all these reasons, it is the right ventricular margin that is usually taken to represent the ventricular septal defect. When there is a valvar orifice overriding the crest of the muscular ventricular septum, the borders of this area do not represent the geometric interventricular communication (see Figure 1). Despite this discrepancy, it is the variations in the anatomy of this right ventricular margin that serve to permit recognition of the phenotypic variations existing amongst patients having the potential for interventricular shunting.

### Ventricular septal defect versus interventricular communication

**Figure 2:**
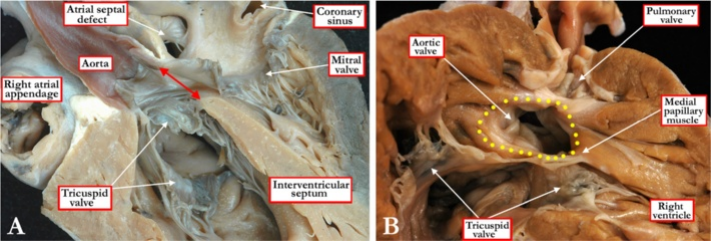
**These images show that the interventricular communication is not necessarily the same thing as the ventricular septal defect.** In Figure 2 **A**, we show a heart with double outlet right ventricle sectioned in four-chamber fashion, showing the aorta arising exclusively from the right ventricle, but with its cranial margin formed by fibrous continuity between the leaflets of the aortic and mitral valves. It is the space between this margin and the crest of the apical muscular septum that is the true interventricular communication. This space (double headed red arrow), however, can never be closed, since such closure would wall off the aorta from the left ventricle. As shown in Panel **B**, in which the free wall of the right ventricle has been lifted away to reveal a defect in a heart with the larger part of the aortic root supported within the right ventricle, in other words effectively a double outlet ventriculo-arterial connection, the outlet septum is exclusively a right ventricular structure, and is fibrous rather than muscular. The yellow dots show the margins of the defect that would be closed so as to place the aortic root in continuity with the cavity of the left ventricle. It is this curved surface that represents the ventricular septal defect, albeit that it is not the geometric interventricular communication.

**Figure 3:**
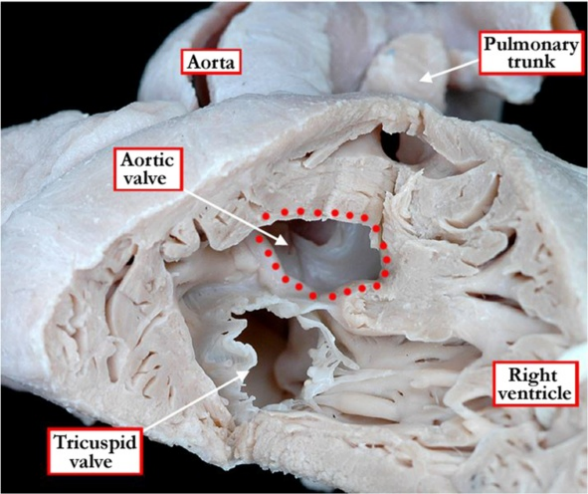
**In this heart, the right ventricle has been windowed, and the structures are viewed from the ventricular apex.** As also seen in Figure 2B, the aortic root overrides the crest of the apical muscular septum, and the greater part of the aortic root is supported within the right ventricle, with the antero-cephalad deviation of the muscular outlet septum producing subpulmonary infundibular stenosis. The specimen shows the tetralogy variant of hearts with double outlet from the right ventricle, but the red dots show the boundaries of the curved surface that is taken to represent the ventricular septal defect.

The right ventricular entrance to the cone of space representing the defect, as shown in Figure 1, is not planar. This is because the crest of the muscular ventricular septum is curved, while the roof of the defect is the portion of the overriding valvar orifice supported by the right ventricle, along with the leading edge of the muscular outlet septum, itself a right ventricular structure by virtue of the valvar overriding. The borders of this defect are those of a curved surface. And, when there is antero-cephalad malalignment of the muscular outlet septum, the curved surface itself is within the cavity of the right ventricle, rather than being strictly interventricular. It is the fact that the patch placed during surgery closes this defect that produces potential semantic problems [6,7]. This is because, as already discussed, holes between the ventricles are called ventricular septal defects in Germanic languages, but as interventricular communications in the Romance languages, such as French, Spanish, Italian, and Portuguese. In terms of geometry, the strict interventricular communication is the space between the ventricles defined on the basis of the continuation of the long axis of the muscular septum (Figure 1A). This communication is much closer to a planar entity, but is different from the curved surface discussed above. In the setting of one of the constellations of lesions that includes a hole between the ventricles, nonetheless, namely double outlet from the right ventricle, it is most frequently this geometric interventricular communication that is nominated as the ventricular septal defect (Figure 2A), rather than the curved surface that incorporates the outlet septum, or its fibrous remnant, as part of its borders (Figure 2B). The outlet septum, be it fibrous or muscular, is of necessity exclusively right ventricular when both arterial trunks arise from the right ventricle. When patients with double outlet right ventricle undergo surgical repair, the hole between the ventricles, or the geometric interventricular communication, is never closed, but rather is tunneled as a part of the surgical correction so as to provide a pathway between the left ventricle and one or other of the subarterial ventricular outlets. Recognition of this fact, however, makes it possible to offer a pragmatic definition for double outlet right ventricle. Should the surgeon consider that, during the operative procedure, he or she has closed the hole between the ventricles, then the patient, prior to repair, must have had concordant or discordant ventriculo-arterial connections. In contrast, if the surgeon considers that he or she has tunneled the hole between the ventricles to one or other subarterial outlet, then the patient must initially have had double outlet right ventricle. Taking account all these points, and for the purpose of the remainder of our review, we take the stance that the curved surface described as the ventricular septal defect is best defined on the basis of its borders as viewed from the cavity of the right ventricle (Figure 3). This means that, in the setting of double outlet right ventricle, it is best to describe the hole as the interventricular communication, rather than a ventricular septal defect.

### Phenotypically different defects

**Figure 4:**
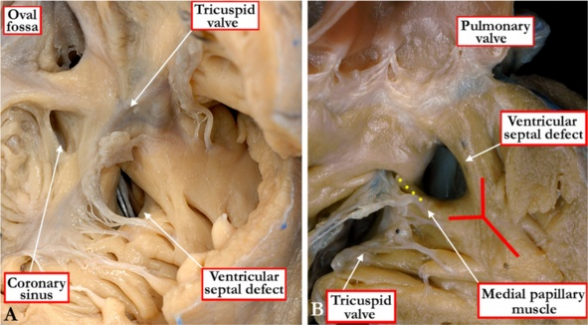
**The view of the opened right atrioventricular junction (A) shows a muscular ventricular septal defect opening to the inlet of the right ventricle beneath the septal leaflet of the tricuspid valve.** In the specimen shown in Figure 4 **B**, the free wall of the right ventricle has been lifted away to show a muscular ventricular septal defect opening into the outlet portion of the right ventricle. The ventricular septal defect lies within the arms of the septal band (red Y), with the caudal arm fusing with the inner heart curvature to produce a muscular bar (yellow dots) that interposes between the leaflets of the atrioventricular and arterial valves.

Although all holes between the ventricles produce the obvious potential for interventricular shunting of blood, not all defects are anatomically the same. The phenotypic differences are important, not only when determining the optimal approach to correction, but also for subsequent genetic counseling. This is because the morphogenesis of the various types is fundamentally different [9]. In terms of phenotypic features, all defects, whether they are isolated or part of a more complex lesion, and when they are viewed from the aspect of the cavity of the right ventricle, can be placed into one of three primary categories [3]. The first category is made up of the holes that have exclusively muscular borders. These holes can have gross malalignment between their caudal and cranial borders. Such malalignment can be described as an additional feature, while recognizing that all defects within this category, when viewed from the right ventricle, have exclusively muscular borders (Figure 4). The defects themselves are described as being muscular. Being encased within the components of the muscular septum, they can open into markedly different parts of the right ventricle. Hence, their geographical location must be described in addition to their anatomic borders as seen from the right ventricle, and in addition to acknowledging the presence of malalignment if present. Their geographical location can be achieved by describing whether they open centrally, apically, anteriorly, or to the right ventricular inlet or outlet components.

**Figure 5:**
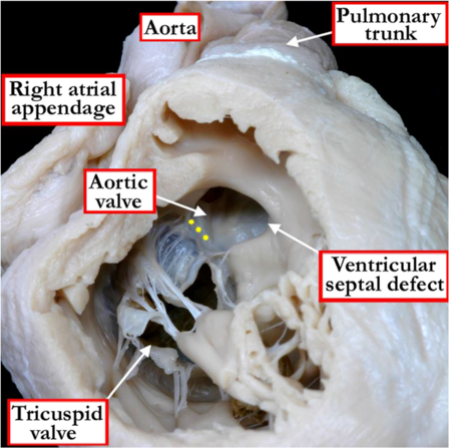
**The right ventricle has been windowed in this heart to show a perimembranous ventricular septal defect opening to the outlet of the right ventricle, with malalignment of the muscular outlet septum so that a small part of the aortic root overrides the crest of the muscular ventricular septum.** The yellow dots mark the fibrous continuity between the leaflets of the tricuspid and the aortic valves, the feature which marks the defect as being perimembranous. Note that, despite the anterior deviation of the muscular outlet septum, which forms the cranial margin of the defect, there is no subpulmonary stenosis. This is an example of the so-called Eisenmenger defect.

The phenotypic feature of the second group, when viewed from the morphologically right ventricle, is that the postero-inferior quadrant of the defect is made up of fibrous tissue. In most instances, the fibrous tissue is itself the continuity between the leaflets of the aortic and tricuspid valves, with the atrioventricular component of the membranous septum included within the fibrous area. The remnant of the interventricular component of the membranous septum is often times seen as a fibrous flap in the postero-inferior corner of such defects. Because of this anatomy, which reflects deficiency of the musculature forming the perimeter of the defect, [7] the lesions are said to be perimembranous (Figure 5) [3]. Should such defects be found in the setting of transposition, it is fibrous continuity between the leaflets of the tricuspid and pulmonary valves that forms the fibrous border. In the setting of double outlet from the right ventricle, and rarely in patients with concordant or discordant ventriculo-arterial connections, the fibrous continuity can be between the leaflets of the mitral and tricuspid valves. These perimembranous defects typically open centrally within the right ventricle, but can extend so that they open primarily to the inlet, then being shielded by the septal leaflet of the tricuspid valve when approached through the right ventricle. They can also be associated with septal malalignment. The malalignment can either be between the atrial and ventricular septal components, or between the apical muscular septum and the muscular outlet septum, or its fibrous remnant (see below).

**Figure 6:**
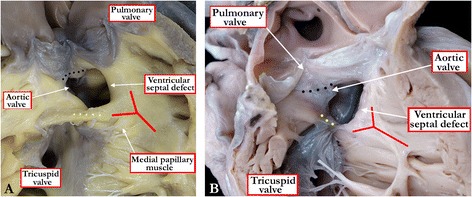
**In this heart (A) with a doubly committed and juxta-arterial ventricular septal defect there is fibrous continuity between the leaflets of the arterial valves (black dots) at the roof of the defect, with a muscular bar (yellow dots) separating the leaflets of the tricuspid and aortic valves.** The muscular bar is formed by fusion of the caudal limb of the septal band (red Y) with the inner heart curvature, the latter also known as the ventriculo-infundibular fold. As shown in Figure 6 **B**, defects with fibrous continuity between the leaflets of the arterial valves (black dots), due to failure of muscularisation of the subpulmonary infundibulum, can also extend to become perimembranous. The leaflets of the tricuspid and aortic valves in this specimen are in fibrous continuity (yellow dots), with the defect again positioned within the Y of the septal band.

The phenotypic feature of the third group is the presence, in the cranial margin of the defect, of fibrous continuity between the leaflets of the aortic and pulmonary valves. This arrangement reflects the failure, during development, to form and muscularise the subpulmonary infundibulum (Figure 6). Because of the absence of any muscular subpulmonary infundibulum, these defects are doubly committed and directly juxta-arterial [3]. Comparable defects are found in the setting of common arterial trunk, and are then positioned directly beneath the leaflets of the common truncal valve. They can also be seen with double outlet from the right ventricle (Figure 2B). Such doubly committed defects usually have a muscular border postero-inferiorly that interposes between the leaflets of the arterial and tricuspid valves (Figure 6A). In a minority of cases, they can extend so as to be bordered postero-inferiorly by fibrous continuity between the aortic and tricuspid valves. In the latter setting, the defects are not only doubly committed and juxta-arterial, but also perimembranous (Figure 6B).

**Figure 7:**
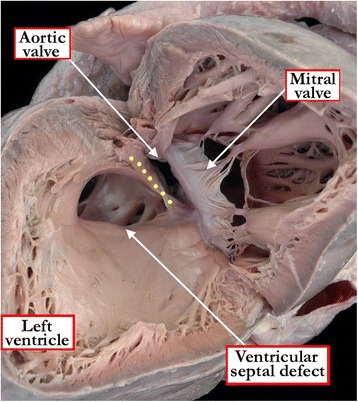
**The view from the left ventricle in this specimen shows a ventricular septal defect with exclusively muscular borders opening towards the outlet of the right ventricle, but with postero-caudal deviation of the muscular outlet septum (yellow dots), causing subaortic stenosis.**

Defects that are perimembranous, as with the muscular defects, can be associated with malalignment of the muscular outlet septum. In the setting of the doubly committed defect, when there is absence of the muscular subpulmonary infundibulum, there can be malalignment of the fibrous remnant of the outlet septum. The malalignment can be in cranial or caudal direction. Cranial malalignment is the essence of the defect seen in tetralogy of Fallot (Figure 3). Such malalignment is also found when perimembranous defects open to the outlet of the right ventricle in the absence of subpulmonary obstruction. This variant is often described as the Eisenmenger defect. Caudal malalignment, in contrast, is typically found when the ventriculo-arterial connections are concordant, in association with either severe aortic coarctation, or interruption of the aortic arch (Figure 7). In the setting of discordant ventriculo-arterial connections, or transposition, caudal malalignment will produce subpulmonary obstruction, whereas extreme cranial malalignment will be associated with severe coarctation or interruption of the aortic arch.

**Figure 8:**
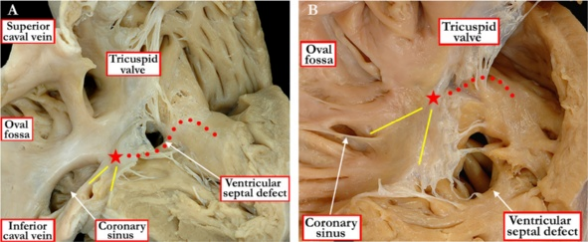
**As shown in these specimens, it is crucial to identify the borders of ventricular septal defects, since this provides information regarding the location of the atrioventricular conduction axis.** As shown in Figure 8 **A**, the axis (red dots) will run along the postero-inferior aspect of perimembranous ventricular septal defects, in other words to the right hand of the surgeon approaching through the tricuspid valve. In contrast, when a muscular defect opens to the inlet of the right ventricle, as shown in Figure 8 **B**, the conduction axis is located to the left hand of the surgeon approaching through the tricuspid valve. The red star shows the location of the atrioventricular node at the apex of the triangle of Koch (yellow lines).

**Figure 9:**
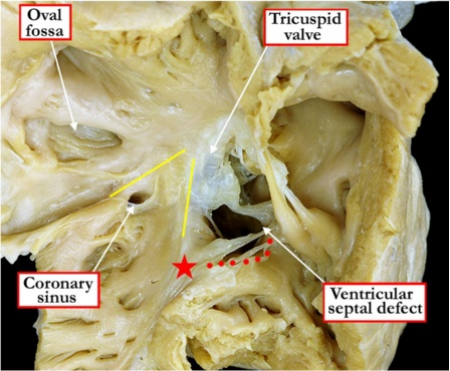
**The rule regarding the location of the conduction axis (red dots) in hearts with perimembranous ventricular septal defects holds good for all instances except when there is malalignment between the atrial septum and the apical muscular septum.** This is associated with straddling and overriding of the tricuspid valve. As shown in the specimen, the conduction axis, carried on the crest of the muscular septum, can no longer take origin from the regular atrioventricular node, which remains at the apex of the triangle of Koch (yellow lines). Instead, it originates from an anomalous node (red star), which is formed at the site of union between the malaligned muscular septum and the inferior atrioventricular junction.

Doubly committed defects, of necessity, open to the right ventricle directly beneath the ventricular outflow tracts. Muscular or perimembranous defects, in contrast, can be positioned so as to open to the inlet, the outlet, or centrally within the right ventricle. With any defect, therefore, it is important to take note of the anatomic nature of its borders when viewed from the right ventricle, and its geographic location relative to the components of the right ventricle. It is the borders that determine the phenotype. The importance of distinguishing the phenotype is exemplified when examining those defects that open to the inlet component of the right ventricle. There is a fundamental difference in the location of the atrioventricular conduction axis when considered in the setting of defects that are perimembranous and open to the inlet, as opposed to those which are muscular [10]. With only one exception, the atrioventricular conduction axis is positioned postero-inferiorly in the setting of perimembranous defects, and to the right hand of the surgeon operating through the tricuspid valve (Figure 8A). When defects open to the inlet of the right ventricle with exclusively muscular borders, then the conduction axis runs antero-cephalad relative to the defect, and is to the left hand of the surgeon working through the tricuspid valve (Figure 8B). The exception to the rule regarding the location of conduction axis in perimembranous defects is found when there is straddling and overriding of the tricuspid valve [11]. In this setting, there is fibrous continuity between the leaflets of the aortic and tricuspid valves, so that the defect itself is perimembranous. The phenotypic feature of these defects, however, is malalignment between the atrial septum and the muscular ventricular septum (Figure 9). Because of this, the atrioventricular conduction axis is unable to take its expected origin from the regular atrioventricular node located within the triangle of Koch. Instead, the conduction axis, which is carried on the crest of the malaligned ventricular septum, takes origin from an anomalous node formed inferiorly in the vestibule of the overriding tricuspid valve. When describing both muscular and perimembranous defects, therefore, it is also necessary to provide information about the way they open to the right ventricle, as well as describing any associated malalignment between the septal components.

Muscular and perimembranous defects can co-exist within the same heart, while muscular defects themselves can be multiple. Indeed, the hardest forms of multiple muscular defects to diagnose and treat are those represented by the so-called “swiss-cheese” septum. These defects almost certainly reflect failure of the septum itself to compact during its prenatal development [9].

### Alternative classifications

**Figure 10:**
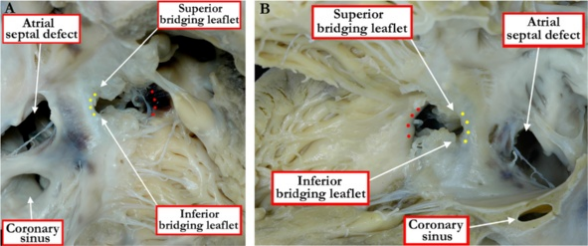
**Defects can also open to the inlet of the right ventricle when they are part of an atrioventricular septal defect with a common atrioventricular valve.** As shown in Figure 10 **A**, when the superior and inferior bridging leaflets are attached to the leading edge of the atrial septum (yellow dots) in this setting, then shunting is possible only at the ventricular level. The ventricular component of the defect (red dots) is then the true ventricular septal defect of atrioventricular canal type. Figure 10 **B** shows the left ventricular view of the same heart, with both bridging leaflets attached to the leading edge of the atrial septum (yellow dots). Note the atrial septal defect within the oval fossa, and the coronary sinus in the left atrioventricular groove.

The account given above is but one of the options available for distinguishing between the different anatomic types of ventricular septal defect. It has the advantage of guiding those closing such defects to the anticipated location of the atrioventricular conduction axis [12]. There are, nonetheless, other popular systems for distinguishing between the different types. A time-honoured system made a distinction between so-called infracristal and supracristal defects, and separated these defects from the muscular ones [13]. The infracristal defects are those now considered to be perimembranous, whereas the supracristal defects are doubly committed and juxta-arterial. Another system used numbers to distinguish between the different types, with so-called Type 1 representing perimembranous defects, Type 2 being muscular, Type 3 being doubly committed, and Type 4 said to represent the inlet defect [14]. The problem with this approach is that, as we have already described, defects opening to the inlet of the right ventricle can be muscular or perimembranous [12]. There is then yet another defect that opens to the right ventricular inlet, namely the atrioventricular septal defect seen in the setting of common atrioventricular junction. In these defects, the bridging leaflets of the common atrioventricular valve are attached to the leading edge of the atrial septum, thus confining shunting at ventricular level [12]. This defect can appropriately be described as a ventricular septal defect of atrioventricular canal type (Figure 10). It has been suggested that the defect seen with straddling tricuspid valve is also an atrioventricular canal type of defect [15]. The hearts with straddling and overriding tricuspid valve, however, have separate right and left atrioventricular junctions. They lack the common junction, which is the essence of the atrioventricular canal malformation [16]. Describing a defect as being “inlet”, therefore, gives no information regarding its phenotypic identity [12]. Within the system we propose, the phenotypic variation will have been documented when diagnosing the entity as being muscular, perimembranous, associated with straddling tricuspid valve, or as being part of an atrioventricular septal defect with common atrioventricular junction [13]. If this information is provided, there will be no need additionally to designate the defect as being of “inlet” variety.

Another popular system accounts for the presence of conoventricular and conal hypoplasia defects [15]. As we understand this approach, conoventricular defects are produced by separation between the muscular outlet, or conal, septum and the remainder of the muscular septum. These lesions can be found with either alignment or malalignment between the conal septum and the apical muscular septum. They can also be found, when viewed from the right ventricle, with partly fibrous or exclusively muscular borders. The defects correlate with those we would describe as being either perimembranous or muscular, and opening either centrally or to the outlet of the right ventricle. The conal hypoplasia defects can have phenotypic features either of muscular defects opening to the right ventricular outlet, or else, with extreme hypoplasia of the subpulmonary conus, they represent the defects which we designate as being doubly committed and juxta-arterial.

### Epidemiology

We have discussed already the reason for underestimating the prevalence of ventricular septal defects. In many patients, the defects are small and the patients asymptomatic. The potential presence of these defects is based on auscultation of a heart murmur. The development, and common use, of cross-sectional echocardiography, and color-flow Doppler interrogation, now permits ready diagnosis of such small defects, which are usually found within the muscular ventricular septum. Those providing the initial estimates of prevalence, however, did not have access to such technology. Another difficulty in providing a precise number for prevalence is that many holes close spontaneously, and thus never come to the attention of physicians. Estimates based on clinical evaluation, [1] therefore, including those based on postmortem examination of specimens, [17] grossly underestimate the true prevalence within the overall population of holes between the ventricles. Evidence of this phenomenon is seen when comparisons are made between the incidence of defects as calculated using postmortem data, [17] and those derived using echocardiographic data. The Baltimore-Washington Infant Study [18], for example, which included echocardiographic examination as a means of diagnosis, revealed a prevalence of muscular defects ten times greater than those noted in previous studies.

As emphasized in our introduction, all studies show that holes between the ventricles in general are found in between one-third and one-half of all patients with congenitally malformed hearts. And, as we have already stated, when considered in isolation, having excluded mitral valvar prolapse and the aortic valve with two leaflets, ventricular septal defects remain the most common congenital cardiac malformations. While the holes between the ventricles can be found in patients with other cardiac anomalies, less than one-twentieth of the patients have chromosomal anomalies, yet ventricular septal defects remain the commonest individual lesion in those patients with abnormal chromosomes. Holes between the ventricles are slightly more common in females than males, albeit the differences in gender proved to be marginal when the estimates were made prior to the availability of echocardiographic diagnosis [19].

### Pathophysiology

The physiologic consequences of any hole between the ventricles are related to its size, and to the relative resistances produced in the pulmonary and systemic vascular beds. Flow to the lungs increases after birth, in keeping with the marked decrease in pulmonary vascular resistance associated with mechanical expansion of the lungs, and exposure of the alveoli to oxygen, which is a potent pulmonary vasodilator. If the defect is large, then the pulmonary flow continues to increase relative to systemic flow concomitant with the regression of the smooth muscle of the intrapulmonary arteries. These changes are associated with the appearance of symptoms after four to six weeks in infants born at term, or after the first two weeks of life, or earlier, in the premature infant. The size of the defect also determines the extent of pulmonary flow, and hence symptoms. If the hole is small, it will be hemodynamically restrictive, thus limiting the size of the left-to-right shunt. If the defect is not hemodynamically restrictive, it will be associated with significant flow to the lungs, and with pulmonary hypertension. Eventually, the increase in pulmonary blood flow, and raised pulmonary pressures, will produce endothelial damage, and permanent changes in pulmonary vascular resistance. When pulmonary vascular resistance exceeds systemic vascular resistance, flow will be from right to left. This is called the Eisenmenger reaction, and will make the patient inoperable. Experience has shown that, to make the distinction regarding restrictive defects, the size of the defect can be related to the dimensions of the aortic root. Our experience suggests that defects half the size of the aortic root, or greater, will produce significant hemodynamic effects, and thus court the risk of producing pulmonary vascular disease. Surgical closure of large and non-restrictive defects can be undertaken at any time after birth in the setting of failure to thrive and symptoms of excessive pulmonary blood flow. In such circumstances, in order to prevent pulmonary vascular disease, the recommendation is usually to close these defects before the infant reaches one year of age.

### Clinical manifestations

The clinical manifestation of an isolated defect is dependent on its pathophysiology. This, again, is related to its size, and the relationship between systemic and pulmonary vascular resistances. As discussed above, it is unusual to find symptoms at birth in infants born with holes between the ventricles. Instead, the symptoms typically become manifest between the ages of 4 and 8 weeks, concomitant with the decrease in pulmonary vascular resistance produced by remodeling of the pulmonary arterioles. Symptoms, however, will occur much earlier in infants born prematurely. Retardation of growth is a major manifestation of the increased flow of blood to the lungs. The increase in the work of breathing, related to the decrease in lung compliance, results in the need for increased caloric intake, which cannot be met during infancy. The increase in flow of blood to the lungs also results in decreased systemic flow, which further compromises the growth failure.

The increase in pulmonary flow, and hence in pulmonary arterial size, causes obstruction in both the large and small airways. It is the anatomic relationship between the pulmonary arteries and left atrium to the tracheobronchial tree that produces the obstruction of the large airways. It is intrapulmonary relationships that create obstruction of the small airways, with resulting pulmonary hyperinflation. The engorgement of the pulmonary arterial circulation may cause pulmonary oedema, and combined with compression of the airways, results in lower airway disease, and produces the symptoms of wheezing, tachypnea, and respiratory distress. If there is associated pulmonary stenosis, however, then flow to the lungs will be decreased. Depending on the extent of the obstruction, this can result in cyanosis.

### Complications

An additional feature complicating some perimembranous defects, but particularly the doubly committed defects, is prolapse of the leaflets of the aortic valve, with ensuing aortic valvar incompetence [20]. Further complications can be produced by development of muscular obstruction in either the right or left ventricular outflow tracts, or development of a fibrous ridge or shelf in the left ventricular outlet. Additional complications relate to the Eisenmenger reaction, and the development in some patients of bacterial endocarditis.

### Diagnosis

**Figure 11:**
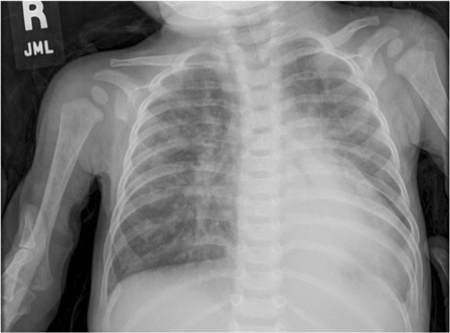
**The chest x-ray, viewed in the antero-posterior projection, from a typical patient with ventricular septal defect shows cardiomegaly, increased pulmonary vascular markings, and atelectasis of the left lower lobe.**

**Figure 12:**
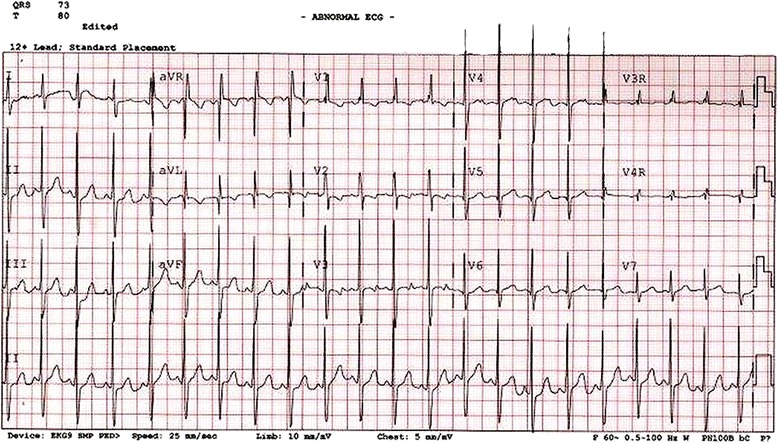
**The electrocardiogram, again from a typical patient with a ventricular septal defect, shows features of biventricular hypertrophy.**

Diagnosis in the past was usually made on the basis of clinical presentation, physical examination, and ancillary studies including chest radiography (Figure 11) and the electrocardiogram (Figure 12). This remains the rule, but today echocardiography plays the major role in diagnosis, and in guiding subsequent decisions regarding management.

The findings at physical examination depend on the size of the defect, along with the changes in pulmonary vascular resistance. In patients with large defects, and low pulmonary vascular resistance, the precordium is hyperactive due to volume and pressure overload of the right ventricle. In such patients, there is a loud second heart sound, with components of both aortic and pulmonary valvar closure. A murmur is also present due to increased pulmonary flow. In patients with large defects, but low pulmonary resistances, the murmur is harsh and holosystolic. A diastolic rumble heard in the mitral area in such patients, due to functional mitral stenosis, will confirm the presence of a large defect. When the pulmonary vascular resistance is increased, however, the second heart sound can be loud and single, and it may not be possible to hear a murmur. It is when the defect is hemodynamically restrictive, and left ventricular pressure greater than the pressure in the right ventricle, that the murmur becomes dependent on the size of the defect. The murmur is typically loud, and often associated with a thrill. If the defect begins to close spontaneously, the murmur will become attenuated.

The chest x-ray is helpful in estimating the flow of blood to the lungs, and hence the significance of the defect. Pulmonary parenchymal findings consistent with increased pulmonary vascular markings are indicative of significant left-to-right shunting, and hence pulmonary over-circulation (Figure 11). Similarly, pulmonary hyperinflation, revealed by trapping of air in the lower airways, is another sign of a significant shunt that may require surgical intervention. Cardiomegaly is the rule in such instances. The electrocardiogram may show features of either left or right ventricular hypertrophy, but often shows features of biventricular hypertrophy (Figure 12).

**Figure 13:**
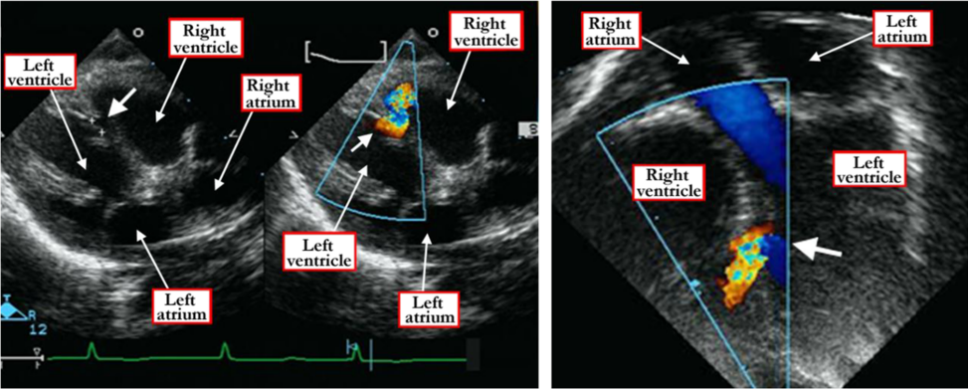
**The cross-sectional echocardiogram, taken in slightly off axis four chamber projections, shows an apical muscular ventricular septal defect (white arrow) in the left hand panel of Figure** 13 **a.** The right hand panel shows the Doppler color flow map of a hemodynamically restrictive muscular ventricular septal defect. The apical five chamber view, shown in Figure 13 **b**, shows the Doppler color flow map of an apical muscular ventricular septal defect (white arrow).

It is the echocardiographic interrogation that now reveals all the information required to answer the questions concerning clinical decision-making, in particular the need for therapeutic closure of the defect. Cross-sectional techniques reveal the anatomic features that distinguish between the muscular, perimembranous, or doubly committed phenotypic variants. The muscular defects are encased within the musculature of the ventricular septum. In some patients with muscular defects, there may be malalignment between the septal components (Figure 13).

**Figure 14:**
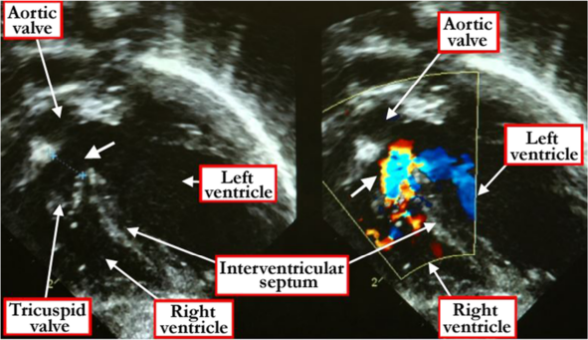
**The subxyphoid four chamber cross-sectional echocardiogram shows the fibrous continuity between the leaflets of the aortic and tricuspid valves that reveals the defect to be perimembranous (white arrow) and opening to the inlet of the right ventricle (left hand panel).** The right hand panel shows the color Doppler flow demonstrating the right-to-left shunt.

The diagnostic feature of the perimembranous defect is the presence of fibrous continuity between valvar leaflets in the postero-inferior quadrant of the defect, with the central fibrous body forming part of the area of continuity (Figure 14). Interrogation from the various echocardiographic windows should show whether muscular or perimembranous defects open centrally, or towards the inlet or outlet of the right ventricle. In the setting of muscular defects, insonation will show whether they are within the middle or apical parts of the ventricular septum. The technique will also reveal the presence of multiple defects.

**Figure 15:**
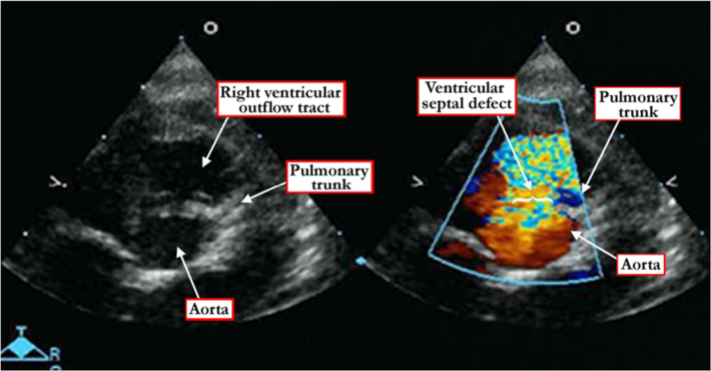
**The short axis echocardiographic cut (left panel) demonstrates the fibrous continuity between the leaflets of the aortic and pulmonary valves that identifies the defect as being doubly committed and juxta-arterial.** The right panel shows the Doppler color flow map of the same defect.

It is the presence of fibrous continuity between the leaflets of the arterial valves in the roof of the defect, and absence of the free-standing muscular subpulmonary infundibulum, which permits the diagnosis of the doubly committed defects (Figure 15). Careful sweeps of the insonating beam will show whether or not such defects are additionally perimembranous.

**Figure 16:**
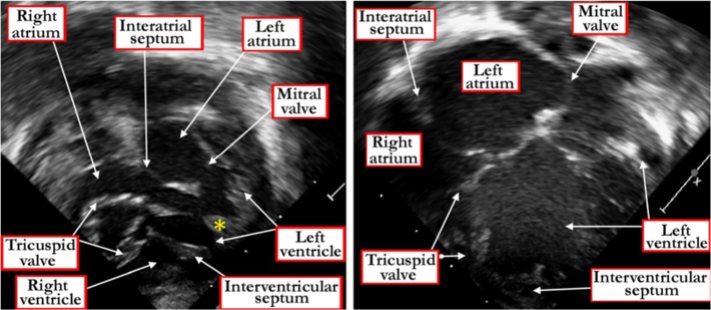
**The panels show subcostal four chamber echocardiographic cuts revealing malalignment between the atrial and muscular ventricular septal structures in a heart with straddling and overriding of the tricuspid valve.** Note the tendinous cords attached to a papillary muscle (yellow asterisk) within the left ventricle. The left hand panel shows the diastolic, and the right panel the systolic location of the overriding leaflets.

Straddling and overriding of the tricuspid valve is revealed, if present, by malalignment between the atrial septum and the muscular ventricular septum (Figure 16). It is important to make this diagnosis because of the abnormal location of the atrioventricular conduction axis (Figure 9).

Spectral Doppler interrogation in the parasternal long axis view is helpful for evaluating the velocity and direction of the blood shunting across the defect. Using this approach, it is possible to calculate the pressure gradient through the defect, this in turn revealing the extent to which the defect is restrictive. Taken overall, the sum of the images determines the location of the defect, and shows its precise relationship to neighbouring cardiac structures. The images also reveal, if present, stenosis or regurgitation of the arterial valves, in particular aortic valvar prolapse. In all instances, it is important to evaluate how the altered hemodynamics caused by the defect affect the size of the left-sided chambers, with dilation of the left atrium and ventricle implying the presence of a large shunt.

Cardiac magnetic resonance imaging and computed tomography will reveal the morphology present, but these techniques are rarely needed once the diagnosis has been made echocardiographically. Cardiac catheterization can also be used to diagnose and delineate the anatomical and hemodynamic characteristics of the holes between the ventricles, including the degree and direction of the net shunting across the defect. Catheterization is particularly useful in those patients with high pulmonary pressures in order to measure the pulmonary vascular resistances. Angiography can also be useful in evaluating the presence of multiple defects. These invasive methods, nonetheless, are usually unnecessary for patients with isolated defects.

Patients are followed on an outpatient basis following the initial physical and echocardiographic evaluation. They can typically be referred, when necessary, for therapeutic closure based exclusively on these techniques.

### Differential diagnosis

**Figure 17:**
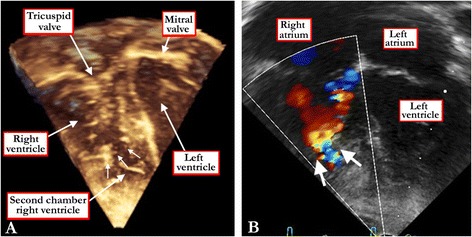
**The three dimensional echocardiographic dataset, shown in four chamber fashion (A), reveals abnormal muscle bundles in the right ventricle (white arrows), producing so-called double chambered right ventricle.** The Doppler color flow map in the same heart **(B)** confirms the presence of the double chambered right ventricle.

**Figure 18:**
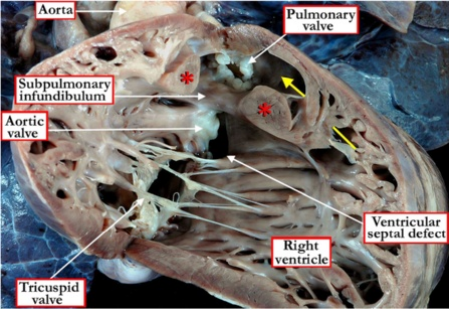
**The free wall of the right ventricle has been lifted from the septal surface to show the features of tetralogy of Fallot with double chambered right ventricle due to anomalous apical septoparietal trabeculations (red asterisks).** The ventricular septal defect is perimembranous. The yellow arrow marks the exit from the apical portion of the right ventricle to the subpulmonary outflow tract. Note the dysplastic and rudimentary leaflets of the pulmonary valve.

Patients with an atrioventricular septal defect can often be differentiated by the presence of the leftward and superior axis on the electrocardiogram. In these patients, shunting may well be confined at ventricular level when the bridging leaflets are attached to the leading edge of the atrial septum (Figure 10). Echocardiography will serve to make the final diagnosis. Patients with double-chambered right ventricle can produce a murmur very similar to that created by flow across a ventricular septal defect. The two-chambered ventricles can also become hypertrophic, with corresponding right ventricular lift and electrocardiographic evidence of hypertrophy. Echocardiographic interrogation should reveal the two chambers, but the divided right ventricle usually co-exists with a ventricular septal defect, so it is also necessary to establish whether there is deficient ventricular septation, and if present, the position and morphology of the defect (Figures 17 and 18). In some cases, measurements of right ventricular pressure, and cineangiography subsequent to catheterization, may be necessary for definitive diagnosis. The features may now better be seen using resonance imaging or computed tomography.

Patients with double outlet right ventricle can present with a large left-to-right shunt and pulmonary over circulation when the interventricular communication is subaortic or doubly committed, and there is no subpulmonary obstruction. If there is overriding of one or other arterial valve in these circumstances, it can be moot as to whether the patient is considered to have double outlet as opposed to a ventricular septal defect, tetralogy of Fallot, or transposition with sub-pulmonary defect. Arbitration should be made on the basis of the proportions of the overriding arterial root supported by the right as opposed to the left ventricles. These features can now be shown with precision using computed tomographic angiography [21]. As we have discussed, a pragmatic distinction can also be made according to whether the surgeon considered it necessary to tunnel one or other arterial valve to the left ventricle, as opposed to simply closing the hole between the ventricles.

Patients with the rare shunt that extends directly from the left ventricular outflow tract to the right atrium can also manifest with a murmur similar that produced by a hole between the ventricles. Indeed, such shunting can be part and parcel of a perimembranous ventricular septal defect, as pointed out by Gerbode and colleagues in their initial description of this lesion [22]. Less frequently, the lesion is due to congenital absence of the atrioventricular component of the membranous septum [22]. When there is shunting to the right atrium, ausculation is more likely to reveal a diastolic component to the murmur, which will radiate to the left mid-sternal border. On chest X-ray or electrocardiogram, the patient will have an enlarged right atrium due to the increased volume coming from the left ventricle. Once again, echocardiographic interrogation should reveal the true anatomic situation.

### Management

In most patients with holes between the ventricles, the defect is sufficiently small to restrict shunting to the extent that there are no symptoms. In such circumstances, additional palliative measures are unnecessary. When interventricular shunting is sufficient to prevent normal growth, producing difficulty in feeding, diaphoresis, or tachypnea, diuretics are the first line of medical palliation. When using diuretics at high doses, note should be taken of the side effects, especially hypokalemia, and a potassium-sparing diuretic used when appropriate. Afterload reduction may also be needed to encourage direct systemic flow from the left ventricle, thereby decreasing the amount of left-to-right shunting through the defect. Afterload reduction is achieved using inhibitors of angiotensin converting enzyme. Inotropy through digoxin is of benefit in those patients with large left-to-right shunts and volume overload of the left ventricle, although its use is increasingly coming under scrutiny. Inotropy and afterload reduction can also be achieved by giving milrinone intravenously, but such therapy is usually reserved for patients awaiting imminent surgery. In general, if a patient is symptomatic and needs palliation, it is preferable to refer for urgent surgical correction.

In patients referred for surgical correction, the defects are almost always closed nowadays by directly placing a patch from the right ventricular side, usually with the surgeon working through the tricuspid valve. It is only patients with large muscular apical defects that are either difficult to see, or to access, from the right ventricular side, or those with the so-called swiss-cheese septum presenting as neonates or infants, who require palliation by banding the pulmonary trunk. The effect of placing the band is to balance the relative pulmonary and systemic resistances, thus minimizing shunting through the defect, and thus protecting the pulmonary vascular bed from over-circulation. When referring patients for surgical correction, care must be taken to ensure that the shunting across the defect is from left-to-right, rather than right-to-left. The latter finding is indicative of so-called Eisenmenger physiology, showing that the pulmonary vascular resistance is so great as to allow decompression of the right ventricle through the defect. Closing such a defect would be detrimental, causing suprasystemic right ventricular systolic pressure, and potentiating the worsening of the pulmonary hypertension responsible for the Eisenmenger condition. It is in these circumstances that cardiac catheterization may be needed to measure with precision the pulmonary arterial pressures.

When closing the defects, the surgeon needs to be aware of the precise location of the atrioventricular conduction axis, which may vary in its relationship to the borders of the defect [10]. In the setting of straddling tricuspid valve, the axis will arise from an anomalous postero-inferior atrioventricular node [11,12]. All the necessary information should now be provided for the surgeon subsequent to echocardiographic interrogation. It should now be exceedingly rare, therefore, for the patient to suffer iatrogenic atrioventricular dissociation, requiring postoperative insertion of a pacemaker [23]. Indeed, the surgical closure of holes between the ventricles should now be accomplished with zero mortality, and minimal morbidity, with the expectation of excellent short and long term outcomes.

It is now also well established that, in the setting of concordant atrioventricular and ventriculo-arterial connections, and in the absence of overriding of the atrioventricular or arterial valves, both muscular and perimembranous defects can be closed percutaneously by insertion of devices using cardiac catheterisation [24,25]. The outcomes subsequent to closure of such “isolated” perimembranous defects have been associated with co-morbidities, including atrioventricular dissociations, as well as interference with neighboring valvar structures. Experience in some centers has revealed iatrogenic heart block after device closure of perimembranous defects to be as high as 22% [26]. Greater success has been achieved subsequent to closure of muscular defects, although muscular defects which are hemodynamically significant are usually found in infants. The smaller size of these patients may make transcatheter closure more technically challenging. Because of this, many centers now advocate using a hybrid approach, inserting devices in the operating room after surgical exposure of the defects.

### Prognosis

The prognosis for the patient with an isolated defect is now excellent. As we have emphasized throughout our review, most patients having muscular ventricular septal defects can anticipate spontaneous closure of the hole. Perimembranous defects can close spontaneously due to apposition of adjacent tissue from the leaflets of the tricuspid valve. It is only doubly committed defects that usually always require closure, since failure to close such defects courts the risk of development of aortic valvar prolapse. Perimembranous or muscular defects co-existing with malalignment of the septal components, nonetheless, will also require surgical attention. Should surgery be indicated, the prognosis for surgical repair is excellent, and most congenital heart surgeons would now expect zero mortality in patients referred after timely diagnosis. The problem still remains, however, for those with significant pulmonary hypertension or Eisenmenger physiology. Prognosis is markedly worse in these instances, and is marked by progressive exercise intolerance, hypoxia, and right ventricular dysfunction. Even with the diagnostic tools that are available today, there will be an occasional patient that presents with an unrepaired or late repaired ventricular septal defect associated with pulmonary hypertension and pulmonary vascular disease. These patients can be referred for evaluation and treatment of evolving pulmonary vasodilator therapy that is now effective in providing stabilization and ameliorating symptoms. Endocarditis is a rare associated problem. When found, the defect is usually hemodynamically restrictive. The substrate is the high velocity jet across the defect creating a Venturi effect, with the subsequent potential for adhesion of platelets, and subsequently vegetations, on the endocardial surface of the defect or the septal leaflet of the tricuspid valve.

## Conclusions

Holes between the ventricles are the commonest lesions found in patients with congenitally malformed hearts. As yet, however, there is no agreement as how best to classify such lesions, nor even on the location of the curved surface that is considered to represent the defect. Based on the review of our own clinical and pathological experiences, we propose that it is the borders of the area taken to represent deficient ventricular septation, as seen from the right ventricle, which should be identified as the defect. When assessed in this fashion, it is then possible to distinguish the phenotypic variants within the group of patients having such lesions on the basis of the anatomic borders of this curved surface, and the fashion in which it opens within the right ventricle. Recognising the variants in this fashion then permits rational analysis of all the clinical features of patients with such deficient ventricular septation in the setting of concordant atrioventricular and ventriculo-arterial connections.
